# Oropouche virus infects primary human intestinal organoids and is inhibited by type I and III interferon treatment

**DOI:** 10.1128/mbio.03003-25

**Published:** 2026-02-05

**Authors:** Xin Wang, Jiajing Li, Alisha Biharie, Annemarie C. de Vries, Marcel J. C. Bijvelds, Harry L. A. Janssen, Wenshi Wang, William M. de Souza, Qiuwei Pan

**Affiliations:** 1 Department of Gastroenterology and Hepatology, Erasmus MC-University Medical Center 6993, Rotterdam, the Netherlands; 2 Toronto Centre for Liver Disease, Toronto General Hospital, University Health Network, Toronto, Ontario, Canada; 3 Department of Pathogen Biology and Immunology, Jiangsu Key Laboratory of Immunity and Metabolism, Jiangsu International Laboratory of Immunity and Metabolism, Xuzhou Medical University, Xuzhou, China; 4 Department of Microbiology, Immunology, and Molecular Genetics, College of Medicine, University of Kentucky 12252, Lexington, Kentucky, USA; Dartmouth College, Hanover, New Hampshire, USA

**Keywords:** Oropouche virus, intestinal organoids, interferons

## Abstract

Oropouche virus (OROV), a neglected arbovirus, has historically been considered a self-limiting infection associated with febrile illness. However, the recent surge in cases since late 2023 has been marked by atypical outcomes, highlighting its underestimated clinical impact. Gastrointestinal symptoms such as diarrhea have also been reported, but the prevalence and mechanistic insight remain largely elusive. Here, through a meta-analysis of 12 identified clinical studies, we revealed a pooled prevalence of diarrhea as 15% (95% CI, 10%–20%) among the Oropouche patient population. In primary human intestinal organoid-based experimental models, we demonstrated productive infection by both a recent patient isolate (OROV-2024) and a historical strain (Be An19991). This is shown by the accumulation of intracellular OROV RNA, release of infectious particles, and immunostaining of OROV glycoprotein Gc. Interestingly, OROV infection mildly triggered the expression of type III interferons, but this endogenous response was insufficient to limit viral replication. In contrast, exogenous treatment with type I and III interferons strongly inhibited OROV replication, with interferon-alpha completely abolishing infectious virus production. Together, these results suggest the human intestine as a potential target organ for OROV infection and highlight interferons as potential therapeutic candidates.

**IMPORTANCE:**

Oropouche virus (OROV) is an emerging arbovirus with rapidly increasing incidence and recent reports of severe disease outcomes. While gastrointestinal symptoms have been described, the intestinal tropism of OROV has not been experimentally explored. By combining meta-analysis of clinical data with human intestinal organoid infection models, we demonstrate that OROV can replicate in intestinal epithelial cells. We further show that, in a human intestinal organoid model, endogenous interferon responses are insufficient to restrict replication, while treatment with interferons exerts potent antiviral activity. These findings highlight the susceptibility of intestinal epithelial cells to OROV infection and the therapeutic potential of interferons.

## OBSERVATION

Oropouche virus (OROV) is a neglected arbovirus transmitted by midges, identified in 1955 in Trinidad and Tobago. OROV has circulated predominantly in the Amazon region in South America (1) and causes Oropouche fever in humans. Since late 2023, OROV has spread beyond the Amazon basin, with a sharp rise in reported cases of Oropouche fever in Brazil, Peru, Colombia, Panama, and Bolivia. Additionally, OROV has been introduced in Cuba, Barbados, and the Dominican Republic (2, 3). OROV travel-associated cases have also been reported in Europe (e.g., Italy, Germany, the Netherlands, and Spain) and North America (Canada and the United States) (4, 5).

**Fig 1:**
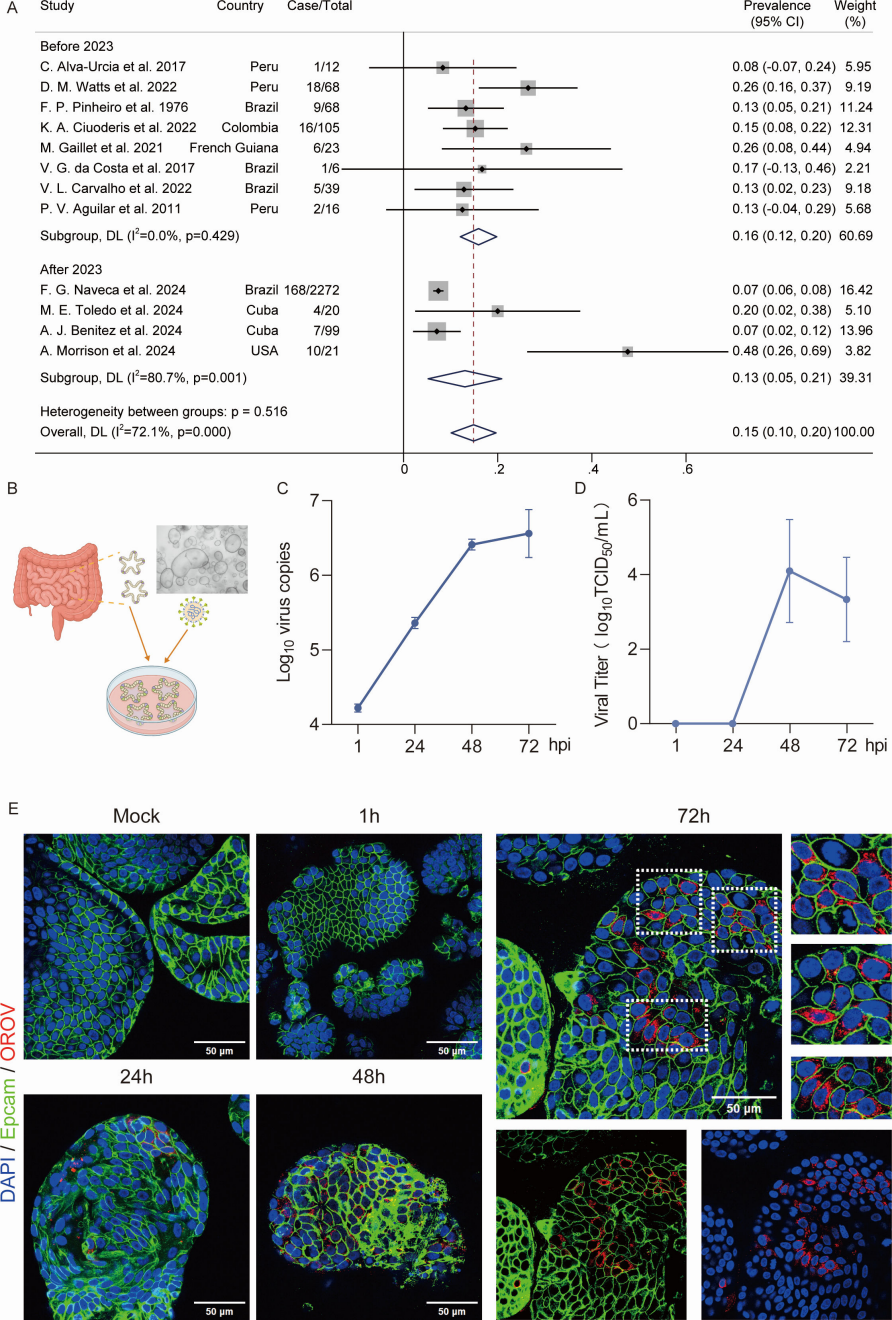
Prevalence of diarrhea in OROV-infected patients and productive infection in human intestinal organoids. (**A**) Pooled prevalence of diarrhea in OROV-infected patients, stratified by publication period. (**B**) Schematic representation of human intestinal organoids inoculated with OROV. A representative bright-field image shows the typical morphology of cultured human intestinal organoids. (**C**) Quantification of viral RNA levels in organoids post-infection with OROV (*n* = 4). (**D**) Quantification of viral titers in culture medium (*n* = 4). (**E**) Representative images of uninfected organoids and OROV-infected organoids at different time points post-infection by immunostaining with the antibodies against OROV Gc glycoprotein (red) and Epcam (green), respectively. DAPI was used to stain nuclei (blue). Scale bar, 50 μm. Data are presented as mean ± SEM.

OROV infection usually results in self-limiting febrile illness, and in some cases, it can lead to severe outcomes, such as vertical transmissions (6) and fatal cases (7). Patients with OROV infection have general symptoms, including fever, myalgia, and headache, similar to dengue fever. Beyond these common symptoms, gastrointestinal manifestations, in particular diarrhea, were also described in clinical reports. To investigate the potential involvement of the intestine in OROV infection, we systematically reviewed the published studies and identified 12 studies meeting the inclusion criteria for meta-analysis (supplemental methods; Fig. S1; Table S2). The pooled prevalence of diarrhea was 15% (95% CI 10%–20%) among OROV-infected patients. Further stratified analysis revealed a prevalence of 16% (95% CI, 12%–20%) before 2023 and 13% (95% CI, 5%–21%) after 2023, among the included studies (Fig. 1A; Fig. S1 and S2; supplemental methods). Heterogeneity among these studies is 72.1% (Fig. 1A). This finding suggests that gastrointestinal involvement is a non-negligible feature of OROV infection and warrants further investigation using physiologically relevant experimental models.

**Fig 2:**
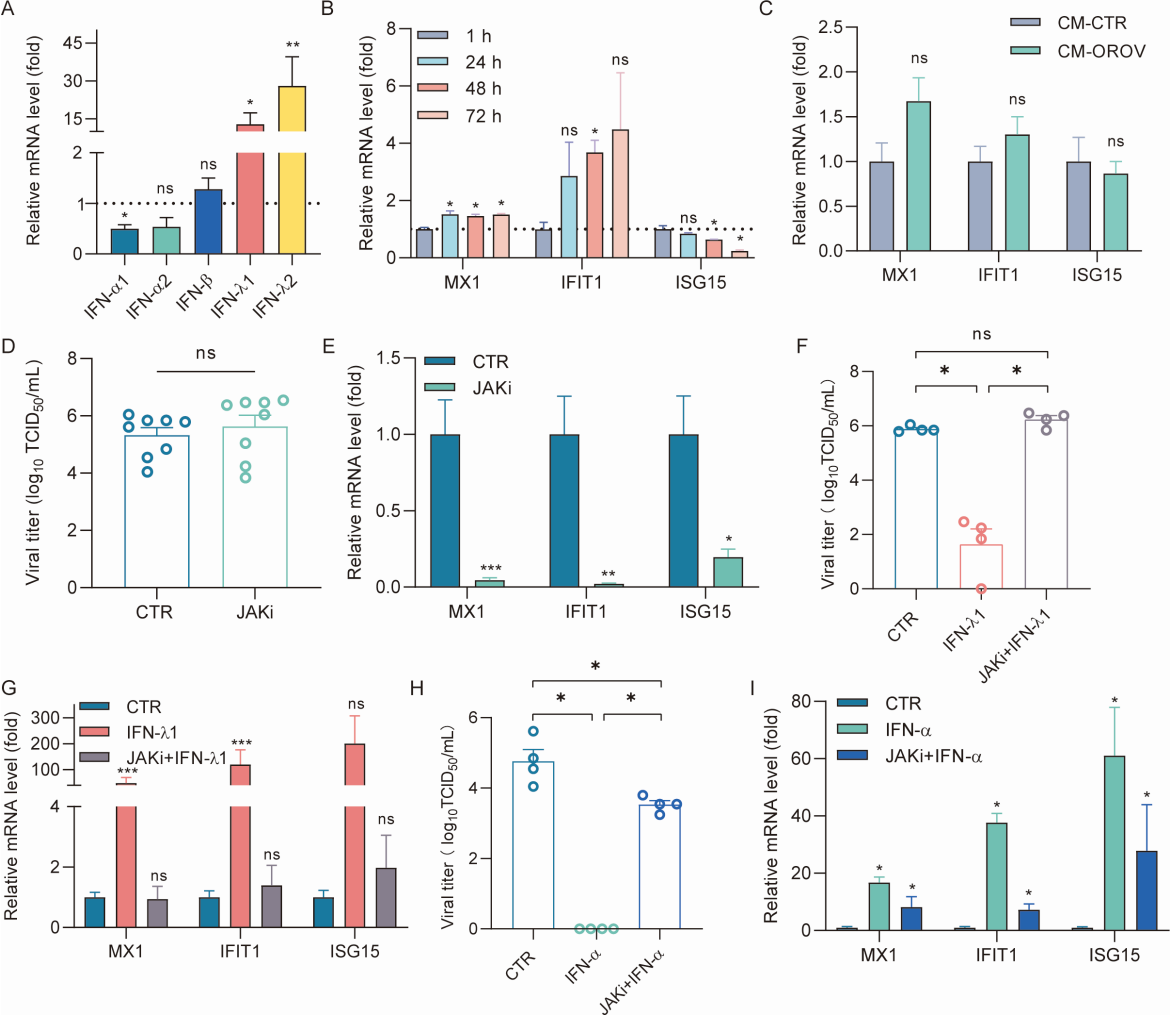
Intrinsic and therapeutic interferon (IFN) responses in human intestinal organoids infected with OROV. (**A**) Relative mRNA levels of type I and III IFNs in OROV-infected compared to uninfected intestinal organoids (*n* = 8). (**B**) Relative mRNA levels of three ISGs, MX1, IFIT1, and ISG15, in OROV-infected compared to uninfected intestinal organoids (*n* = 4). (**C**) Relative mRNA of MX1, IFIT1, and ISG15 in human intestinal Caco2 cells treated by culture medium harvested from uninfected intestinal organoids (CM-CTR; set as 1) and OROV-infected intestinal organoids (CM-OROV) for 24 h (*n* = 7). (**D and E**) Intestinal organoids were infected with OROV and then treated with 10 µM JAK inhibitor 1 (JAKi). (**D**) Viral titers were determined by TCID_50_ assay (*n* = 8), and (**E**) relative mRNA levels of MX1, IFIT1, and ISG15 in organoids were analyzed by qRT-PCR (*n* = 8). (**F–I**) Intestinal organoids were infected with OROV and then treated with 1,000 IU/mL IFN-λ1 or IFN-λ1 plus 10 µM JAKi, or IFN-α, or IFN-α plus 10 µM JAKi. (**F and H**) Viral titers were determined by TCID_50_ assay (*n* = 4). Relative mRNA levels of MX1, IFIT1, and ISG15 in organoids treated with IFN-λ1 (**G**, *n* = 7) or IFN-α (**I**, *n* = 4) were analyzed by qRT-PCR. Data are presented as mean ± SEM; *P < 0.05; **P < 0.01; ***P < 0.001; and ns, not significant.

To investigate whether human intestinal epithelium is susceptible to OROV infection, we used human intestinal organoids as an *in vitro* model and cultured them as we described previously (Fig. 1B) (8, 9). Organoids were infected with the OROV strain IRCCS-SCDC_1/2024 (OROV-2024), which was isolated in 2024 from an imported case in Italy (4) and is a representative strain of the currently circulating epidemic. Quantifying viral genome copies in infected organoids by qRT-PCR (supplemental methods; Table S1) showed that intracellular viral RNA copies increased over time, rising from 4.2 log_10_ at 1 h post-infection to 6.5 log_10_ at 3 days post-infection (Fig. 1C). Consistent with the intracellular OROV genome detection, infectious OROV particles in the culture medium were detectable at 48 h post-infection, reaching titers of 4 log_10_ TCID_50_/mL (Fig. 1D). Consistently, we found similar results when inoculating organoids with the OROV strain Be An19991, a Brazilian OROV prototype isolated in 1960 (Fig. S3A and B). Next, to visualize the infection, immunostaining of the OROV surface glycoprotein Gc was performed. We detected the presence of OROV in intestinal organoids at 24 h post-infection, and it increased in a time-dependent manner, reaching a peak at 72 h post-infection. As expected, Gc protein is undetectable in uninfected organoids (Fig. 1E; Fig. S3C). Altogether, primary human intestinal organoids are susceptible to OROV infection and support viral replication.

Interferons (IFNs) act as a first line of defense against viral infection through the induction of IFN-stimulated genes (ISGs) (10). Type I and type III IFNs, including IFN-α and IFN-λ, respectively, are potent antiviral cytokines induced by infections with non-redundant functions (11). To investigate whether OROV infection in intestinal organoids can trigger IFN response, we quantified gene expression of the key members of type I and III IFNs at 72 h post-infection. There appears to be a mild elevation of type III but not type I IFN expression (Fig. 2A). Quantification of the expression of three representative ISGs indicated a modest increase in IFIT1 expression, but not in MX1 and ISG15 expression (Fig. 2B). To functionally validate whether OROV infection triggers the production of notable levels of IFNs, we harvested conditioned medium from OROV-infected and uninfected organoids at 72 h post-infection, which was subsequently applied to treat human intestinal Caco2 cells. qRT-PCR analysis demonstrated that the induction of ISG expression in Caco2 cells is minimal by treatment with conditioned medium from OROV-infected organoids (Fig. 2C). Furthermore, treatment with a JAK inhibitor, which potently inhibits IFN response and ISG expression, did not facilitate the OROV infection in intestinal organoids (Fig. 2D and E). Previous studies, primarily in mouse models, have shown that type III IFNs play an important role in controlling infections locally at the intestinal barrier surfaces and are often induced in these epithelial cells by the invading pathogens (12). However, in our human intestinal organoids, there is minimal induction of IFN response by OROV with no clear contribution to limit the infection. Although the underlying mechanisms of these disparities require further investigation, a previous study has reported that the OROV nonstructural protein NSs is a type I IFN antagonist (13). In addition to NSs, like other bunyaviruses, the OROV polymerase is predicted to contain an OTU domain that may also assist immune evasion via deubiquitinating and deISGylating activities (14). Nevertheless, OROV remains capable of inducing type I IFN production in immune cells (15, 16), and elevated serum concentrations of IFN-α have been observed in infected patients (17).

Recombinant IFN-α has been used for decades in clinics, whereas IFN-λ has been extensively studied in clinical trials to treat viral infections (18, 19). To evaluate the therapeutic potential of IFNs to treat OROV infection, we treated infected organoids with IFN-λ1 or IFN-α. This significantly decreased the production of infectious OROV particles, which can be reverted by the JAK inhibitor (Fig. 2F). ISG expression was induced by IFN-λ1 (Fig. 2G). Importantly, treatment with IFN-α completely inhibited the production of infectious OROV particles in infected organoids, which can be substantially (although not completely) reverted by the JAK inhibitor (Fig. 2H). Consistently, IFN-α triggered the induction of ISG expression (Fig. 2I).

In summary, this study revealed a pooled prevalence of 15% of diarrhea following OROV infection in patients. These findings suggest that gastrointestinal manifestations should not be overlooked in clinical diagnosis and management of OROV infection. Using an organoid model, we further demonstrated that OROV is capable of productively infecting intestinal epithelial cells, suggesting it as a potential cause of intestinal manifestations. How the virus reaches the intestinal tract during natural infection remains unclear. Possibilities include systemic dissemination, interactions with infected immune cells, and in regions with inadequate sanitation infrastructure, direct fecal-oral transmission cannot be excluded. More evidence is needed to validate these hypotheses.

Although there is minimal impact of the intrinsic IFN produced by intestinal epithelial cells in controlling OROV infection in intestinal organoids, treatment with recombinant IFNs profoundly inhibited OROV infection. Of note, a previous study in a mouse model has demonstrated that pre-treatment with IFN-α exerted some anti-OROV activities, but treatment initiated post-infection had no inhibitory effects (20). Finally, our results derived from an innovative human-based model strongly support further exploration of IFNs as a therapeutic option for treating OROV infection.

Of note, this study has some limitations. First, only symptom-based clinical data were available for our meta-analysis. Patient-derived samples will be needed to directly confirm intestinal involvement during OROV infection in future research. Second, although human intestinal organoids capture key epithelial features, they do not fully represent the cellular complexity of the intestinal environment, particularly the contribution of immune cells. Thus, the IFN responses observed in this study require further validation *in vivo*, which will help to better assess its therapeutic potential. Finally, more comprehensive studies integrating clinical investigation, broader viral lineage diversity, and advanced models will be essential to better define gastrointestinal manifestations of OROV infection.

## SUPPLEMENTAL MATERIAL

Supplemental file mbio.03003-25-s0001.pdf Supplemental methods, figures, and tables.

ASM does not own the copyrights to Supplemental Material that may be linked to, or accessed through, an article. The authors have granted ASM a non-exclusive, world-wide license to publish the Supplemental Material files. Please contact the corresponding author directly for reuse.
