# Supplementary material for: Oropouche virus infects primary human intestinal organoids and is inhibited by type I and III interferon treatment
Source: mBio. 2026 Feb 5;17(3):e03003-25. doi: 10.1128/mbio.03003-25 (PMC12977569; doi:10.1128/mbio.03003-25)
Supplement: Supplemental file — Supplemental methods, figures, and tables. [file mbio.03003-25-s0001.pdf]

Supplementary information

Supplementary Methods

Systematic review and meta-analysis

Search strategy

An exhaustive search strategy was developed by an experienced information specialist (M.F.M.) in cooperation with one of the investigators (J.L.). The search was developed in Embase.com, optimized for sensitivity and then translated to other databases following the method as described by Bramer et al.(1) The search strategies for Medline and Embase used relevant thesaurus terms from Medical Subject Headings (MeSH) and Emtree respectively. In both databases, terms were searched in titles, abstracts and author keywords. Terms were combined with Boolean operators AND and OR and proximity operators were used to combine terms into phrases. Search results are shown in Table S2. The references were imported into EndNote and duplicates were removed by an experienced information specialist (M.F.M.) using the method as described by Bramer et al.(2)

Embase 373

('Oropouche orthobunyavirus'/exp OR (oropouche*):ab,ti,kw)

Medline 326

(Oropouche orthobunyavirus.rs. OR (oropouche*).ab,ti,kf.)

Cochrane 0

((oropouche*):ab,ti,kw)

Web of Science 333 + 16

TS=(oropouche*)

Global Index Medicus

oropouche

bioRxiv 63

oropouche

medRxiv 21

oropouche

Google Scholar

oropouche virus|fever|viral|outbreak|orthobunyavirus|bunyavirus|epidemic

Selection criteria

Studies with original data were included in terms of the following criteria: cross-sectional or longitudinal observational studies with available data from oropouche virus (OROV) infected cases in febrile patients or local general population. Study without OROV infection and case reports were excluded.

Data collection

Two investigators (X.W. and J.L.) extracted data from each included study based on a standardized form. Study characteristics included first author, country and study period. Study population data contained total sample size and OROV infected cases proved by virus or antibody detection. Demographic characteristics (age and sex) and clinical signs and symptoms of OROV infected cases were also collected.

All statistical analyses were implemented by STATA 15.0, with a p value of 0.05 or less considered statistically significant. A random-effects (DerSimonian and Laird) meta-analysis model was used for all analyses to generate pooled estimates, reported as prevalence with the corresponding 95% confidence interval (CI), based on evidence suggesting more robust effect estimates with random-effects models compared to fixed-effects models.(3, 4) Heterogeneity between studies was assessed using Cochrane Q and I^2 statistic, with an I^2 of at least 50% considered to be significant heterogeneity.(5) Quality assessment of included articles was assessed by Joanna Briggs Institute Critical Appraisal Checklist.(6) Potential publication bias was mainly assessed with the Egger's test and the

funnel plots (only for more than 10 studies) of the study size against transformed outcome values (e.g., log transformation for prevalence).(7)

Reagents

Human IFN-α (Merck) and IFN-λ1 (Invitrogen) were dissolved in PBS with the stock concentration of $1x10^7$ IU/mL and $1x10^6$ IU/mL, respectively. JAK inhibitor 1 (Bio-Connect BV) was dissolved in DMSO with a final concentration of 5 mg/mL.

Intestinal organoids culture

Human primary intestinal organoids were isolated and cultured as we previously described (8). The use of human intestinal tissue for research purpose including culturing into organoids was approved by the Medical Ethical Council of the Erasmus MC, and informed consent was given (MEC-2021-0432; MEC-2023-0629). These organoids were cultured in organoid expansion medium (OEM), based on advanced DMEM/F12 (Invitrogen), supplemented with 1% penicillin/streptomycin (Life Technologies), 10 mM HEPES, 1xGlutamax, 1 mM N2, 1 mM B27 (all from Invitrogen), 1 µM N-acetylcysteine (Sigma) and the following growth factors: 50 ng/L mouse epidermal growth factor (mEGF), 50% Wnt3a-conditioned medium (WCM) and 10% noggin-conditioned medium (NCM), 20% Rspo1-conditioned medium, 10 µM nicotinamide (Sigma), 10 nM gastrin (Sigma), 500 nM A83–01 (Tocris) and 10 µM SB202190 (Sigma). The medium was refreshed every 2-3 days, and organoids were passaged 1:3 every 5–7 days.

OROV strains and viral inoculation

The historical OROV strain BE AN 19991 was obtained from YARU/A.Travassos, Inst. Evandro Chagas, Belem, Brazil and virus stocks was prepared in Vero E6 cells. OROV strain IRCCS-SCDC_1/2024_OROV-2024 (OROV-2024) was isolated from an imported case in Italy (9). For infection, human intestinal organoids were mechanically fragmented and then exposed to

OROV at a dose of 10^6 $TCID_{50}$ per ~10,000 organoids for 2 hours at 37°C. To enhance the infection efficiency, the virus-organoid suspension was gently resuspended every 30 minutes during the incubation. Subsequently, fragmented organoids were centrifuged at 300 g for 5 minutes at 4°C, and the supernatant was discarded. Organoids were then washed three times with advanced DMEM/F12 to remove residual viruses, embedded in Matrigel (Corning), and cultured in OEM. Each experimental group included at least four biological replicates, defined as independent organoid cultures.

Treatment

After virus-inoculated organoids were embedded in Matrigel. IFNs or JAKi diluted in OEM were added to the organoids. After treating for 72 hours, samples were harvested for following assays.

Cell culture

Vero E6 cells and Caco2 cells were maintained in Dulbecco's modified Eagle medium (DMEM; Lonza) supplemented with 10% fetal calf serum (Hyclone) and 100 U/mL penicillin-streptomycin. All cell cultures were confirmed to be mycoplasma negative through regularly testing by GATC Biotech (Konstanz).

Culture supernatant harvest and treatment

Culture supernatant of OROV-infected or -uninfected organoids were harvested and then filtered by 0.2μm filter. Filtered medium was mix with equal volume of DMEM and then added to Caco2 cell. After 24 hours, cells were harvested for following assays.

Immunofluorescence assay

Organoids were fixed in 4% paraformaldehyde solution at 4°C overnight. The slides containing organoids were then rinsed three times with phosphate-buffered saline (PBS) for 5 minutes each time, followed by permeabilizing with PBS containing 0.2% (v/v) Triton X-100 for 15 minutes. Then, the slides were twice rinsed with PBS for 5 minutes, followed by

incubation with blocking solution (5% donkey serum, 1% bovine serum albumin, and 0.2% Triton X-100 in PBS) at room temperature for 1 hour. Next, the slides were incubated in a humidity chamber with primary antibody diluted in blocking solution at 4°C overnight. Primary antibodies used in this study are as follows: Anti-Oropouche orthobunyavirus Gc (provided by Dr. Sven Reiche from Federal Research Institute for Animal Health, Germany) and anti-Epcam (Abcam). Slides were washed three times for 5 minutes each in PBS before 1-hour incubation with secondary antibodies. Nuclei were stained with 4',6-diamidino-2-phenylindole (Invitrogen). Images were obtained using Leica SP5 cell imaging system.

RNA extraction and RT-qPCR detection

Total RNA was extracted using the Macherey-Nagel NucleoSpin RNA II Kit (Bioke) and quantified by Nanodrop ND-1000 (Wilmington). cDNA was synthesized using a cDNA synthesis kit (Takara Bio). Viral DNA levels were quantified by SYBR Green-based RT-qPCR (Applied Biosystems SYBR Green PCR Master Mix; Thermo Fisher Scientific) with the QuantStudio Real-Time PCR Systems (Applied Biosystems by Thermo Fisher Scientific). Relative gene expression was normalized to GAPDH using the formula $2^{-\Delta\Delta CT}$ ($\Delta\Delta CT = \Delta CT_{sample} - \Delta CT_{control}$). Primers used for quantitative reverse transcription PCR (qRT-PCR) were synthesized by Sigma-Aldrich. The primers used in this study are provided in supplementary Table 1.

Quantification of virus genome copy numbers

To determine viral genome copy numbers, cDNA from OROV infected organoids was used as template to generate the insert DNA, which was then cloned into the vector to generate the plasmid containing OROV S segment sequence. The plasmid was serially diluted 10-fold from 10^2 to 10^8 copies and quantified by RT-qPCR to establish a standard curve. The linear regression equation was determined as $Ct = -3.323 \times \log_{10}[copies] + 35.47$, with an R^2 of 0.9989. Based on the standard deviation of the residuals (Sy.x = 0.2406), the limit of detection (LOD) and limit of quantification (LOQ) were calculated as 1.65 copies and 5.31

copies per reaction, respectively. These correspond to Ct values of approximately 34.9 (LOD) and 33.1 (LOQ).

$TCID_{50}$ Assay

Infectious OROV titers were determined using a 50% tissue culture infectious dose (TCID50) assay. Briefly, serial ten-fold dilutions of harvested supernatant were inoculated onto monolayers of Vero cells seeded at a density of 2,000 cells per well in 96-well plates. Plates were incubated at 37 °C for 3-4 days and monitored daily for cytopathic effect (CPE) using light microscopy. The $TCID_{50}$ value was calculated by using the Reed-Muench method.

Statistical analysis

Statistical analysis was performed using GraphPad Prism8 statistics software (GraphPad, San Diego, USA). All data are presented as mean ± standard error of the mean (s.e.m.). Comparison between two groups was analysed by Mann–Whitney U test. For longitudinal data, a mixed-effects model (restricted maximum likelihood, REML) was used to assess the effects of time, virus strain, and their interaction. Asterisks indicated the degree of significant differences compared with the controls (*$P < 0.05$, **$P < 0.01$, ***$P < 0.001$).

Supplementary figures

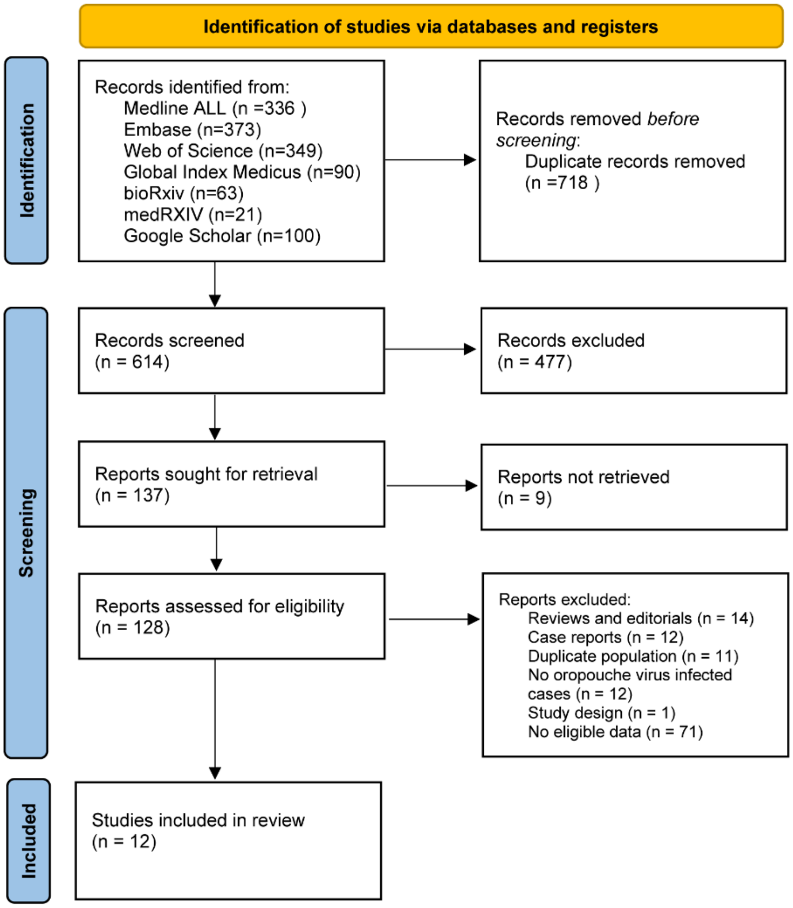

Figure S1. Flow diagram for study selection

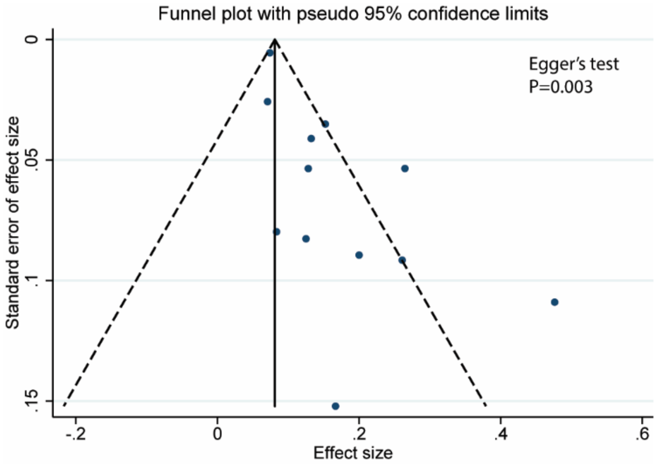

Figure S2. Funnel plot of the studies included in the meta-analysis.

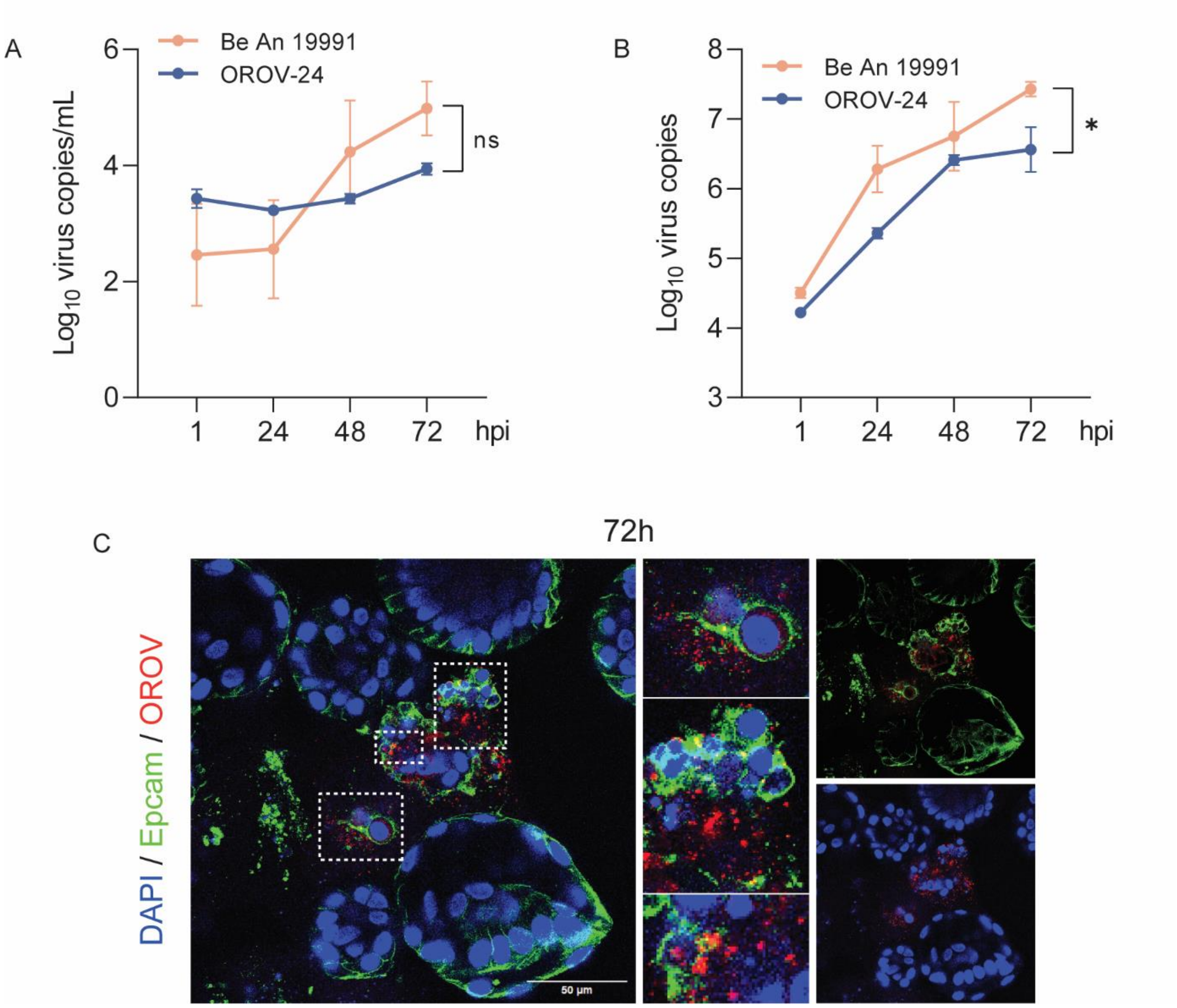

Figure S3. Productive infection of OROV in human intestinal organoids.

(A) Quantification of viral RNA levels in culture medium post-infection with Be An 19991 and OROV-24 strain (n = 4). (B) Quantification of viral RNA levels in organoids post-infection with Be An 19991 and OROV-24 strain (n = 4). (C) Representative images OROV-infected organoids at 72 h post-infection by immunostaining with the antibodies against OROV Gc glycoprotein (red), and Epcam (green), respectively. DAPI was used to stain nuclei (blue). Scale bar, 50 μm; h: hour; hpi: hour post-infection. Data are presented as mean ± SEM; statistical analysis by mixed-effects model with REML; *$P < 0.05$; ns, not significant

Supplementary table

Supplementary table 1. Primer sequences

Name	Sequence
OROV-F	GACAAGTSCTCAATGCTGGTGT
OROV-R	CGTTGTCCGGSACTGGATT
GAPDH-F	GTCTCCTCTGACTTCAACAGCG
GAPDH-R	ACCACCCTGTTGCTGTAGCCAA
IFN-α1-F	AGAAGGCTCCAGCCATCTCTGT
IFN-α1-R	TGCTGGTAGAGTTCGGTGCAGA
IFN-α2-F	TGGGCTGTGATCTGCCTCAAAC
IFN-α2-R	CAGCCTTTTGGAACTGGTTGCC
IFN-β1-F	CTTGGATTCCTACAAAGAAGCAGC
IFN-β1-R	TCCTCCTTCTGGAACTGCTGCA
IFN-λ1-F	GGAAGACAGGAGAGCTGCAACT
IFN-λ1-R	AACTGGGAAGGGCTGCCACATT
IFN-λ2-F	TCGCTTCTGCTGAAGGACTGCA
IFN-λ2-R	CCTCCAGAACCTTCAGCGTCAG
IFIT1-F	GCCTTGCTGAAGTGTGGAGGAA
IFIT1-R	ATCCAGGCGATAGGCAGAGATC
MX1-F	GGCTGTTTACCAGACTCCGACA
MX1-R	CACAAAGCCTGGCAGCTCTCTA
ISG15-F	CTCTGAGCATCCTGGTGAGGAA
ISG15-R	AAGGTCAGCCAGAACAGGTCGT

Supplementary table 2. Overview of article search

Database searched	Platform	Years of coverage	Records	Records after duplicates removed
Medline ALL	Ovid	1946 - Present	336	333
Embase	Embase.com	1971 - Present	373	73
Web of Science Core Collection*	Web of Knowledge	1975 - Present	333	60
Web of Science Preprint Citation Index	Web of Knowledge		16	3
Cochrane Central Register of Controlled Trials*	Wiley	1992 - Present	0	0
Global Index Medicus	www.globalindexmedicus.net		90	71
bioRxiv	www.biorxiv.org		63	52
medRxiv	www.medrxiv.org		21	10
Additional Search Engines: Google Scholar** (100 top-ranked)			100	12
**Total**			**1332**	**614**

*Science Citation Index Expanded (1975-present) ; Social Sciences Citation Index (1975-present) ; Arts & Humanities Citation Index (1975-present) ; Conference Proceedings Citation Index- Science (1990-present) ; Conference Proceedings Citation Index- Social Science & Humanities (1990-present) ; Emerging Sources Citation Index (2005-present)

**Google Scholar was searched via "Publish or Perish" to download the results in EndNote.

No other database limits were used than those specified in the search strategies

Supplementary table 3. Characteristics of included studies

Study	Country	Study period	Virus detection	Sample size	Virus-proved cases	Antibody detection	Sample size	Antibody-proved cases	Quality assessment
F. P. Pinheiro et al. 1976	Brazil	From February to April 1975	Virus isolation	243	68	Hemagglutination-inhibiting against the Be An 19991 and IgM capture ELISA	243	18	6
C. Alva-Urcia et al. 2017	Peru	From January to March 2016	RT-PCR	139	12				5
V. G. da Costa et al. 2017	Brazil	From 2011 to 2013				IgM EIA-ICC	130	6	5
M. Gaillet et al. 2021	French Guiana	From August 11 to October 15 2020	RT-PCR	41	11	Neutralization	41	16	6
V. L. Carvalho et al. 2022	Brazil	From January to February 2018	Virus isolation	26	14	IgM capture ELISA	90	36	5
K. A. Ciuoderis et al. 2022	Colombia	During 2019-2022	RT-PCR	791	87	IgM capture ELISA	503	27	6
D. M. Watts et al. 2022	Peru	From October 1, 1993 to September 30, 1999				IgM ELISA	6607	68	6
P. V. Aguilar et al. 2011	Peru	In 2006				Neutralization	1037	154	5
M. E. Toledo et al. 2024	Cuba	From May 27, 2024 to June 2, 2024		3393	20				6
F. G. Naveca et al. 2024	Brazil	From 2022 to 2024	RT-PCR		2272				6
A. J. Benitez et al. 2024	Cuba	In 2024	RT-PCR	120	99				5
A. Morrison et al. 2024	USA	In 2024	RT-PCR		21	Neutralization		21	5

References

1. Bramer WM, de Jonge GB, Rethlefsen ML, Mast F, Kleijnen J. 2018. A systematic approach to searching: an efficient and complete method to develop literature searches. J Med Libr Assoc 106:531-541.
2. Bramer WM, Giustini D, de Jonge GB, Holland L, Bekhuis T. 2016. De-duplication of database search results for systematic reviews in EndNote. J Med Libr Assoc 104:240-3.
3. Bell A, Fairbrother M, Jones K. 2019. Fixed and random effects models: making an informed choice. Quality & Quantity 53:1051-1074.
4. Tufanaru C, Munn Z, Stephenson M, Aromataris E. 2015. Fixed or random effects meta-analysis? Common methodological issues in systematic reviews of effectiveness. Int J Evid Based Healthc 13:196-207.
5. Melsen WG, Bootsma MC, Rovers MM, Bonten MJ. 2014. The effects of clinical and statistical heterogeneity on the predictive values of results from meta-analyses. Clin Microbiol Infect 20:123-9.
6. Moola S MZ, Tufanaru C, Aromataris E, Sears K, Sfetcu R, Currie M, Qureshi R, Mattis P, Lisy K, Mu P-F. 2020. Chapter 7: Systematic reviews of etiology and risk. *In* Aromataris E MZ (ed). JBI, JBI Manual for Evidence Synthesis.
7. Hunter JP, Saratzis A, Sutton AJ, Boucher RH, Sayers RD, Bown MJ. 2014. In meta-analyses of proportion studies, funnel plots were found to be an inaccurate method of assessing publication bias. J Clin Epidemiol 67:897-903.
8. Yin Y, Bijvelds M, Dang W, Xu L, van der Eijk AA, Knipping K, Tuysuz N, Dekkers JF, Wang Y, de Jonge J, Sprengers D, van der Laan LJ, Beekman JM, Ten Berge D, Metselaar HJ, de Jonge H, Koopmans MP, Peppelenbosch MP, Pan Q. 2015. Modeling rotavirus infection and antiviral therapy using primary intestinal organoids. Antiviral Res 123:120-31.
9. Deiana M, Malagò S, Mori A, Accordini S, Matucci A, Passarelli Mantovani R, Gianesini N, Huits R, Piubelli C, Gobbi FG, Capobianchi MR, Castilletti C. 2024. Full Genome Characterization of the First Oropouche Virus Isolate Imported in Europe from Cuba. Viruses 16.
